# Network diffusion-based analysis of high-throughput data for the detection of differentially enriched modules

**DOI:** 10.1038/srep34841

**Published:** 2016-10-12

**Authors:** Matteo Bersanelli, Ettore Mosca, Daniel Remondini, Gastone Castellani, Luciano Milanesi

**Affiliations:** 1 Department of Physics and Astronomy, Universita’di Bologna, Bologna, Via B. Pichat 6/2, 40127, Italy; 2 Institute of Biomedical Technologies, National Research Council, Segrate (MI), via Fratelli Cervi 93, 20090, Italy

## Abstract

A relation exists between network proximity of molecular entities in interaction networks, functional similarity and association with diseases. The identification of network regions associated with biological functions and pathologies is a major goal in systems biology. We describe a network diffusion-based pipeline for the interpretation of different types of omics in the context of molecular interaction networks. We introduce the network smoothing index, a network-based quantity that allows to jointly quantify the amount of omics information in genes and in their network neighbourhood, using network diffusion to define network proximity. The approach is applicable to both descriptive and inferential statistics calculated on omics data. We also show that network resampling, applied to gene lists ranked by quantities derived from the network smoothing index, indicates the presence of significantly connected genes. As a proof of principle, we identified gene modules enriched in somatic mutations and transcriptional variations observed in samples of prostate adenocarcinoma (PRAD). In line with the local hypothesis, network smoothing index and network resampling underlined the existence of a connected component of genes harbouring molecular alterations in PRAD.

Cellular functions are carried out by modules of interacting molecular entities^1^. Complex intracellular circuits can be modelled as networks in which vertices are molecular entities and links are (direct and indirect) interactions among entities. According to the so-called local hypothesis, functional similarity is related to network proximity and, in line with it, the molecular entities involved in the same disease have an increased tendency to interact with each other^2^. This knowledge, in combination with the growing availability of molecular interactions data, offers the opportunity to develop computational approaches that use network proximity as a tool to predict molecular species function and disease association^3^,^4^.

More generally, the definition of the network regions associated with biological functions and diseases is a major goal in systems biology^2^,^5^. Several integrative approaches, which jointly analyse interactions and molecular profiles, have been proposed^6^,^7^,^8^,^9^,^10^,^11^,^12^ and were recently classified into four broad categories: identification of active modules, identification of conserved modules across species, identification of differential modules and identification of composite modules^13^. However, the identification of modules is still an open challenge in bioinformatics research. First of all, the size of biological networks makes the search for subnetworks time-consuming. Secondly, technological biases in high-throughput approaches for interaction detection and molecular profiling can compromise analyses accuracy. Thirdly, datasets collected with different omics platforms (e.g. microarrays, sequencing, mass spectrometry) vary considerably in type, distribution of values and completeness. Lastly, our biological knowledge is still limited: just to mention two relevant examples, according to recent estimates, only the 10% of protein-protein interactions (PPIs) may be known^14^ and even if more than half of all proteins are glycosylated, knowledge about the glycosylation process is still limited^15^. Another challenging aspect is that while topological communities often represent functional modules, they do not overlap with disease modules: therefore, the search for disease subnetworks can not be faced using only community detection methods^2^,^6^.

Recently, network diffusion-based approaches, which simulate the diffusion of a quantity throughout a network in order to calculate a global measure of network proximity, have been successfully proposed in several applications, taking advantage of the local hypothesis. A few examples are the association of genes and protein complexes with diseases^16^, the stratification of tumour mutations^7^, the identification of biomarkers in genome-wide studies^17^,^18^ and the study of virus-host molecular interactions^19^,^20^.

Examples of network diffusion-based bioinformatics tools include NBS^7^, HotNet^8^, TieDie^10^, ResponseNet^11^, RegMod^12^ and stSVM^21^. NBS^7^ smooths somatic mutations profiles and then uses network-based non-negative matrix factorisation to stratify subjects. Hotnet^8^ uses statistics derived from somatic mutations as input for a diffusion process in order to identify active network regions. TieDie^10^ and ResponseNet^11^ use two different approaches to find the subnetwork that connects two sets (sources and targets) of network vertices, which can represent genomic perturbations and gene expression variations. TieDie^10^ uses a diffusion approach to find a subnetwork of sources, targets and (predicted) linkers that are “logically consistent” in relation to their molecular profiles. ResponseNet^11^ formulates a minimum-cost flow optimisation problem that is solved by linear programming. RegMod^12^ was proposed to find disease-associated modules using interactions and gene expression data; this approach uses the support vector regression method with a diffusion kernel in order to find active modules. stSVM smoothes a vector of *t* statistics by means of a random walk kernel and uses a support vector machine to select a set of significant genes^21^.

In this paper, we describe a network diffusion-based pipeline for the identification of network regions carrying the most significant molecular alterations measured with different types of omics platforms (Fig. 1). We show that the network smoothing index (*S*), a network diffusion-based quantity introduced here, is a simple and informative measure to jointly quantify the amount of omics information associated with a molecular entity (e.g. gene, mRNA, protein) and the information in network proximity to it. Consequently, we describe two general applications of *S* for finding differentially enriched regions, in relation to the type of input statistics *S* is derived from: the variation of *S* between two sets of samples (Δ*S*) or the permutation-adjusted *S (Sp*) for, respectively, descriptive statistics or inferential statistics. We also describe a procedure (network resampling) for the assessment of the presence of significantly connected components among entities ranked by Δ*S* or *Sp*.

We show the performance of network diffusion, Δ*S*, *Sp* and network resampling in a simulated dataset. Then, as a proof of principle, we apply these tools to spot PPI network regions differentially enriched in somatic mutations (SM) and gene expression (GE) variations between two prognostic groups of patients affected by prostate adenocarcinoma (PRAD). We carry out the analyses using five datasets of molecular interactions.

The strategy described here can be in principle applied to the analysis of any high-throughput dataset that can be mapped to a network of interactions. We implemented the pipeline used in our study into an R package (http://www.interomics.eu/tools).

## Results

### Identification of differentially enriched modules

**Figure 1:**
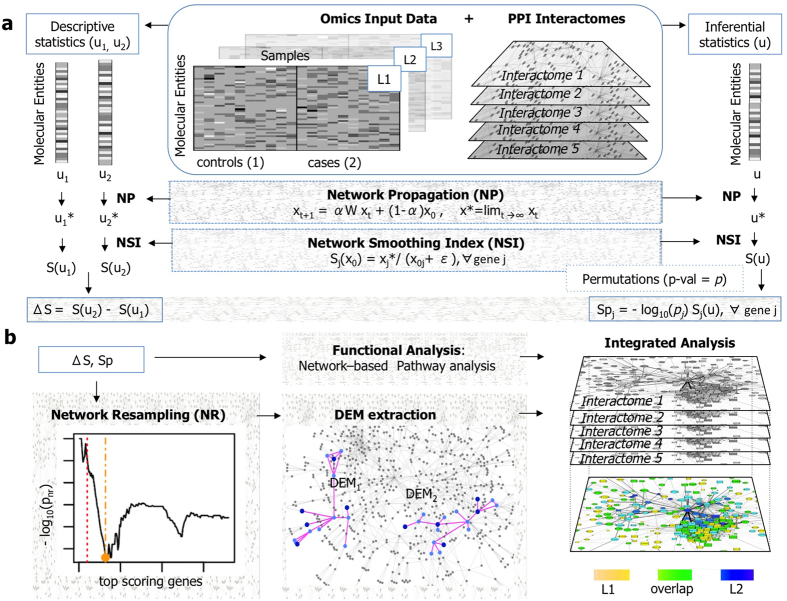
Network diffusion-based analysis of omics for the identification of differentially enriched network regions. (**a**) Statistics (descriptive on the left, inferential on the right) of molecular profiles are smoothed by means of network propagation and the network smoothing index is computed. (**b**) Identification of significantly connected components among genes ranked by Δ*S* or *Sp*, and network-based functional analysis.

Network diffusion methods can be applied to different types of initial quantities, like genes-by-subjects matrices^7^ and summary statistics^9^,^10^,^21^. Such differences are mainly motivated by the type of input data, the objective of the analysis and the algorithm used to generate the results. We consider to apply network diffusion on two types of input: descriptive statistics that summarise the information of a sample group (Fig. 1a left-hand side) and inferential statistics that describe molecular variations between two classes (Fig. 1a right-hand side).

In relation to the physical model of diffusion we used, we refer to the positive elements of the input as “sources” and to the represented molecular quantity as information or fluid. Network diffusion allows to “smooth” the information associated with molecular entities according to a given pattern of interactions, encoded in the adjacency matrix **A**, a square binary matrix where positive elements *a*_*ij*_ indicate the presence of an interaction between entities *i* and *j*. We consider the diffusion method designated as “network propagation”^16^ to smooth any input statistic *x*_0_:


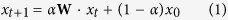


where **W** is a symmetrically normalised version of **A** (see methods) and 0 < *α* < 1 controls the contribution of the two addends. At each iteration *t*, the amount of information in each vertex is the sum of its initial information and the total amount of information associated with its neighbours at the previous iteration. This iterative procedure will converge in a finite number of iterations to a particular state x^*^ 22. Note that we can interpret the iterative procedure of Equation (1) as a diffusion process in which a fluid enters from sources, flows through the links between vertices and exits at a constant first order rate from each vertex. In particular, after a proper rescaling, network propagation is equivalent to the laplacian dynamics of the open system of type *dx*/*dt* = −*L*′*x* + *b*, where *L*′ = *I* − *αW* and *b* represents the molecular profile. This equivalence implies that the steady state reached by the laplacian dynamics is the same state x^*^ to which Equation (1) converges (Supplementary Note). At steady state, high values are associated with sources and with vertices in network proximity to sources. Note that network diffusion, in contrast to other methods, is a global measure of network proximity, i.e. it considers the whole network^4^.

In order to quantify the average amount of information at steady state (x^*^) in relation to the initial one (*x*_0_) in a sample group, we introduce the network smoothing index *S*_*j*_ of a molecular entity *j*:


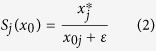


where *ε* is a parameter that weights the relative importance of initial and final states. Small values of *ε* underline the gain of information in relation to the initial state, while when *ε* → ∞ only the final state 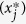 matters. A reasonable compromise can be found in order to prioritise both sources and entities in network proximity to sources (see below the results on PRAD data).

At this point, network smoothing indices calculated on descriptive statistics relative to cases (*u*_2_) and controls (*u*_1_) can be subtracted:


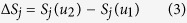


where the Δ*S*_*j*_ jointly quantifies the differential amount of molecular variation observed in entity *j* and in its neighbourhood between two sample groups. Note that the calculation of Δ*S*_*j*_ contrasts the effect of hubs that assume high *S*_*j*_ in both groups only because of their centrality. In other words, since the topology of the network is the same for the two sample groups, the effects ascribable only to topology are mitigated.

If the network smoothing index is obtained from a inferential statistics (*u*), then permutations can be used to mitigate the effect of hubs. In this case, we define the *Sp* value for each gene *j*:


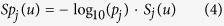


where *p*_*j*_ is the fraction of times an *S*_*j*_ obtained from the smoothing of a randomised version of (*u*) is equal or greater than the real *S*_*j*_. The quantities *S*, Δ*S*, *Sp*, *u*, *u*_1_ and *u*_2_ are vectors of length equal to the entire number of molecular entities considered.

At this point, the top molecular entities sorted in decreasing order of Δ*S* (or *Sp*) belong to regions with a differential content of information. In order to identify one or more differentially enriched modules we need to cut this list and extract the subnetworks composed of such top entities. Accordingly, we define the non-decreasing objective function Ω for molecular entities ranked by, for example, Δ*S*:


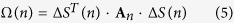


where **A**_*n*_ is the adjacency matrix for only the first *n* top scoring molecular entities. In other words, the function Ω(*n*) is the sum of all the products Δ*S*_*i*_Δ*S*_*j*_ between the pairs (*i*, *j*) of interacting (*a*_*ij*_ = 1) molecular entities. According to the local hypothesis^2^, if the difference between the two classes is the consequence of an underlying biological function or pathobiological process, we should expect a significant pattern of connections among the molecular entities with the highest Δ*S*^6^. In order to quantify such significance, for each rank *n*, we calculate the values of Ω(*n*) using *k* resampled adjacency matrices, where we randomly assign the existing links among vertices conserving the same degree distribution. Then, we calculate the corresponding network resampling *p* values (*p*_*nr*_), which are equal to the mean number of times a random assignment of the links among the first *n* molecular entities determines a value of Ω(*n*) higher than or equal to the one observed with real links (Supplementary Note and Fig. S1). Following this procedure, the ranks associated with low values of *p*_*nr*_ indicate the presence of connected genes with top Δ*S* scores, or *Sp* in case Ω(*n*) was calculated on a list ranked by *Sp*.

### Performance on simulated data

**Figure 2:**
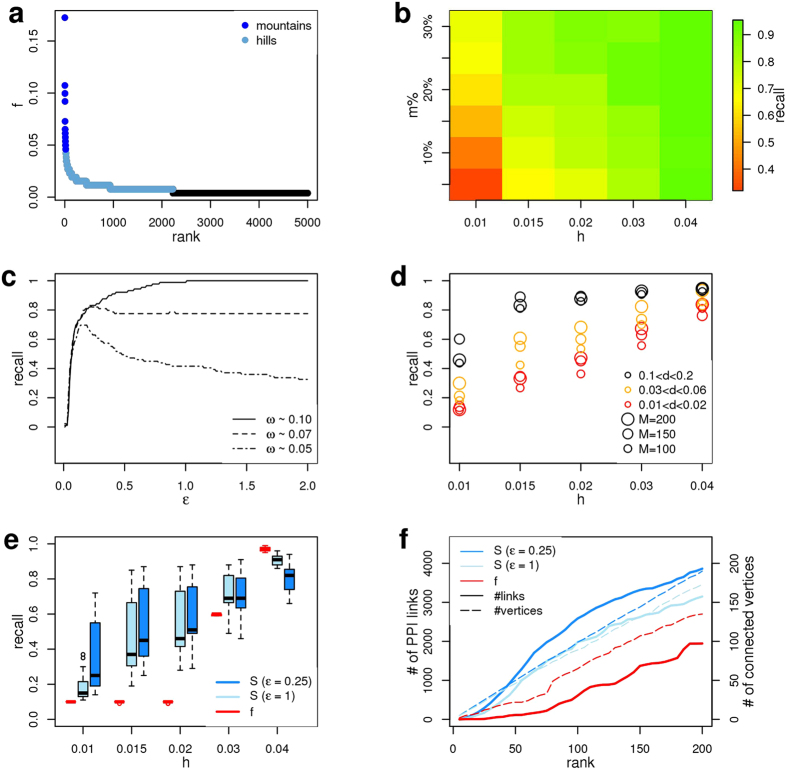
Performance of differential network smoothing index in simulated datasets containing gene modules enriched in omics information. (**a**) Somatic gene mutation relative frequencies ranked in decreasing order to underline mountains and hills. (**b**) Heatmap of recall values with varying percentage of mountain genes *m*% and average hill frequency *h*, calculated on a sample of modules of size *M* = 100. (**c**) Fraction of recalled genes for different values of parameter *ε* with varying *ω*. (**d**) Average recall obtained on several toy datasets of different sizes *M* = {100, 150, 200} and topological density *d*, for different values of *h*. (**e**) Comparison between recall values obtained ranking genes by *S* or *f*, for different values of *ω*, on several toy datasets. (**f**) Number of links and number of connected genes among the top ranking genes ranked by *f* or *S*. (**a–f**) Simulations were run using STRING PPIs. (**c–f**) The signal *ω* was computed with *m*_%_ = 0.1.

We designed a series of simulated datasets to study the ability of the network smoothing index to prioritise genes belonging to network regions (shortly modules) with a higher content of omics information in comparison to the rest of the network. We considered the generic definition of modules as random subnetworks, where the existence of a finite path that connects each pair of module genes is the only topological requirement, because disease proteins do not necessarily reside within locally dense communities^6^ and, more generally, it is not clear to which extent functional modules, topological modules and disease modules overlap^2^. We associated with each module a specific amount of signal (*ω*) non-uniformly distributed among the genes, in order to have a few module genes contributing to the most of the signal and all the other module genes with lower or not significant amounts of signal (Fig. 2a) (see methods). This distribution was inspired by what is observed in real datasets, like the “mountains” (highly mutated genes) and “hills” (genes altered infrequently) observed in cancer mutation landscape^23^. Moreover, it models a more general scenario in which the alteration of some module genes is observed in many individuals (higher signal), other module genes are altered more specifically (lower signal) and, lastly, some module genes are marginally altered. Conversely, outside the module the signal was randomly distributed. We used STRING^24^ and PRAD SM data (from TCGA^25^) as sources, respectively, of molecular interactions and biological signal (see methods). The simulated datasets were defined such that the real amounts of mutation per patient and per gene were not modified.

We explored several configurations, varying *ω*, the distribution of *ω*, the parameter *ε* (Equation 2), module size and module topological density (the number of existing links over all possible links among the module genes). For each configuration we computed *S* and calculated the recall as the fraction of module genes that appear among the top *M* genes ranked in decreasing order of *S*, where *M* is module size.

We observed high recalls either in modules enriched in mountains and in those enriched in hills (Fig. 2b). When the biological signal is particularly high, the best performance is obtained for high values of *ε*, while when the module is composed of a mixture of genes with strong and marginal variation, we have observed the maximum recall for smaller values of *ε (ε* ≈ 0.25) (Fig. 2c). The performance of *S* increases with increasing topological density (Fig. 2d). This behaviour is particularly evident for low values of *ω*, underlying that a high density of connections strengthens the ability of the index to prioritise genes in network proximity to those with a high content of molecular alterations. The use of *S* determines higher recall than the network-free quantity *f* (gene mutation relative frequency), apart from the extreme case in which the module genes are exactly the top genes ranked by *f* (Fig. 2e). *S* determined the identification of more connected network regions with a higher content of module genes compared to what we observed using *f* (Fig. 2f).

**Figure 3:**
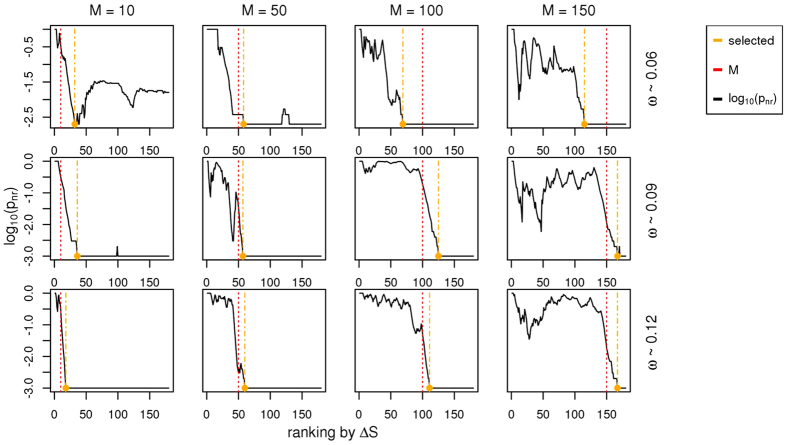
Identification of significantly connected genes with network resampling *p* values in simulated datasets containing a gene module enriched in omics information. Network resampling *p* values (*p*_*nr*_) calculated for each rank of gene lists ordered by decreasing values of Δ*S* in datasets containing gene modules of different size (red lines, *M*) and signal (*ω*). Yellow lines indicate the smallest ranks associated with the presence of significantly connected components. Simulations were run using STRING PPIs.

We assessed the ability of network resampling in predicting module size. We ordered genes by Δ*S*, which quantified the difference between a simulated dataset with a gene module enriched in biological information (as described above) and a simulated dataset without such enrichment. Also in this case, the real amounts of mutation per patient and per gene were not modified. As the signal *ω* increases, the size of significantly connected components approaches module size (Fig. 3). However, network resampling correctly highlighted module size also for lower values of *ω*. Note that we expected some discrepancy between predicted and real module size due to the module definition procedure itself (e.g. *ω* enriched in a module subregion by the random assignment procedure).

### Prostate adenocarcinoma

As a proof of principle, we applied the network-based pipeline to the identification of molecular interaction networks enriched in genes with a higher content of SMs and GE differences between two distinct PRAD prognostic grade groups, G5 and G2, where the higher the grade the poorer the prognosis. We used these datasets to illustrate two possible types of input data. In particular, in the case of SM data, we calculated the relative frequency of gene mutation within each prognostic group (*f*), obtaining two vectors of descriptive statistics (*f*_1_, *f*_2_), and the variation between these two (Δ*f*). We applied network diffusion to *f*_1_ and *f*_2_, calculated the corresponding *S*_1_, *S*_2_ and Δ*S*. In the case of GE data, we calculated an inferential statistics between G5 and G2 (*lfcp*), which combines absolute gene log fold change and adjusted *p* value of a moderated *t* statistics. We applied network diffusion to *lfcp*, considering as input the binary vector in which positive elements are the top 500 genes with the highest *lfcp*, calculated the corresponding *S* and *Sp*. We repeated these analysis using five collections of direct (physical) and indirect (functional) PPIs (see methods).

**Figure 4:**
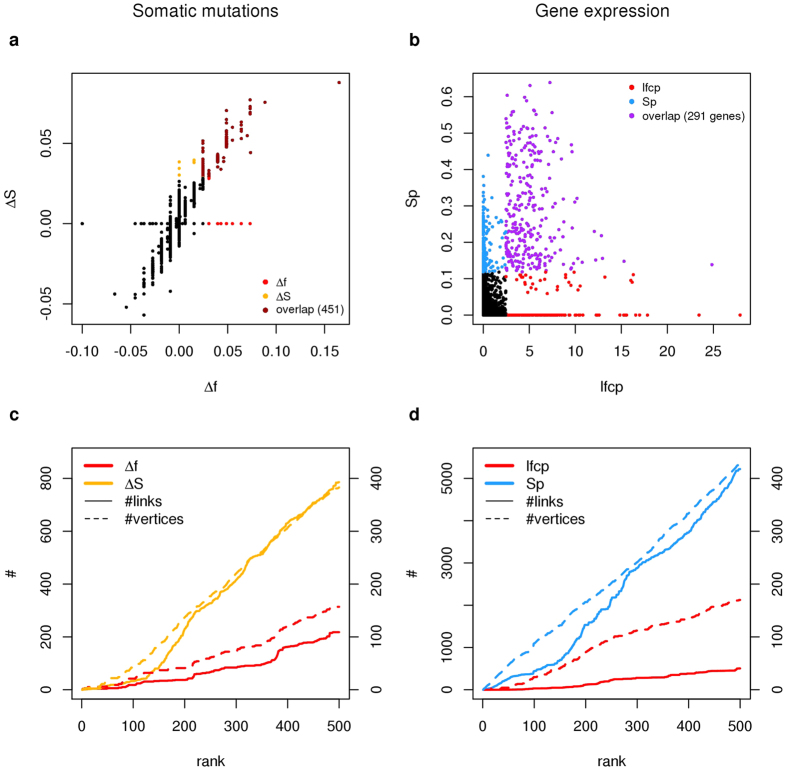
Comparison of network-based and network-free quantities calculated on somatic mutation and gene expression data from PRAD samples associated with two different prognostic groups. (**a,b**) Scatter plot with network-based (*y*–axis) *vs* network-free (*x*–axis) gene scores calculated on PRAD SM (**a**) and GE (**b**) data; colours indicate the top 500 genes ranked by network-free (red) or network-based (yellow, blue) scores and the overlaps (brown, purple). (**c,d**) Number of links (*y*–axis, left) and number of connected genes (*y*–axis, right) within the first 500 genes ordered by network-based (Δ*S*, *Sp*) and network-free (Δ*f*, *lfcp*) gene scores, calculated on PRAD SM (**c**) and PRAD GE (**d**) data. (**a–d**) Δ*S* and *Sp* were calculated using STRING PPIs and, respectively, *ε* = 0.25 and *ε* = 1. (**c,d**) ^#^Number of links (vertical axis, left) or number of veritces (vertical axis, right).

Of course, genes for which no interaction information is available in the considered interactome do not have a network-based value (Δ*S* = *Sp* = 0 in Fig. 4a,b). As expected, Δ*S* and *Sp* prioritised genes jointly considering the relevance of the “network-free” statistics associated with each gene and the network-free statistics of genes in network proximity to each gene (Fig. 4a,b). Genes with the highest variations of Δ*f* or *lfcp* are also associated with the highest values of Δ*S* or *Sp* respectively. This overlap can be tuned using the parameter *ε* (Supplementary Figs S2–3). Genes with similar values of Δ*f* or *lfcp* are discriminated in relation to their network location: the higher the network proximity of a gene to other genes associated with relevant Δ*f* or *lfcp*, the higher its Δ*S* or *Sp* respectively. As a consequence, top ranking genes ordered by Δ*S* and *Sp* are more connected and form bigger networks than genes ordered by network-free quantities (Fig. 4c,d). Despite the differences in terms of number of proteins and interactions, we observed these results in all interactomes (Supplementary Figs S4–7).

**Figure 5:**
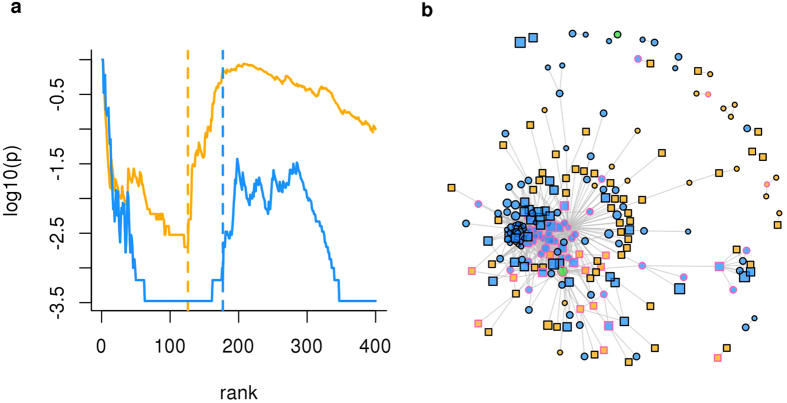
Gene modules enriched in genes with different somatic mutations and gene expression levels between two PRAD prognostic groups. (**a**) *p*_*nr*_ value of gene lists ranked by Δ*S* (SM, yellow) and *Sp* (GE, blue); vertical lines indicate the top ranking genes selected to be part of the corresponding gene modules. (**b**) Network of genes belonging to SM module (yellow), GE module (blue) or both (green); square/circle = the gene is/is not ranked by network-free statistics within the first *M* positions (*M* = module size); vertex size = the larger the size the higher the gene score (maximum between Δ*S* and *Sp*); pink border = genes that occur in at least 10 articles on PRAD (Supplementary Data S1–2). These results were obtained with STRING PPIs.

We applied the network resampling procedure to genes ranked by decreasing values of Δ*S* (enrichment of SM in G5 in comparison to G2) and *Sp* (enrichment in GE variations between G5 and G2), and found significantly connected modules in both cases (Fig. 5a). SM gene modules range from 109 and 231 genes depending on the interactome (Table 1). A total of 342 distinct genes occur in these modules while 45 genes occur in all of them. Similarly, the GE gene modules range from 100 to 351 genes, with a total of 518 distinct genes and 33 found in all interactomes (Table 1).

In addition to associated with the most extreme molecular variations between G5 and G2 (and therefore highly ranked also by network-free approaches) these modules contain genes specifically prioritised by Δ*S* and *Sp*. SM and GE modules contain genes that are highly cited in the literature of PRAD, some of which were specifically prioritised using networks (Table 2). The two genes TP53 and CDK2, the expression of which do not vary significantly, are examples of highly ranked genes because of their network proximity to differentially expressed genes (GE data), while, analogously, MEFV and TRPS1 are two examples of genes specifically found using networks in the analysis of SM data (Table 2). Other genes are not part of the current PRAD literature, but could be interesting candidate for further studies, since are in network proximity to genes with molecular alterations and/or already associated with the pathology (Supplementary Data S1–2). Even if only a few genes belong to both SM module and GE module (e.g. TP53 and ANO4 using STRING, Supplementary Data S1–2), several molecular interactions exist among genes of the two modules (Fig. 5b).

**Figure 6:**
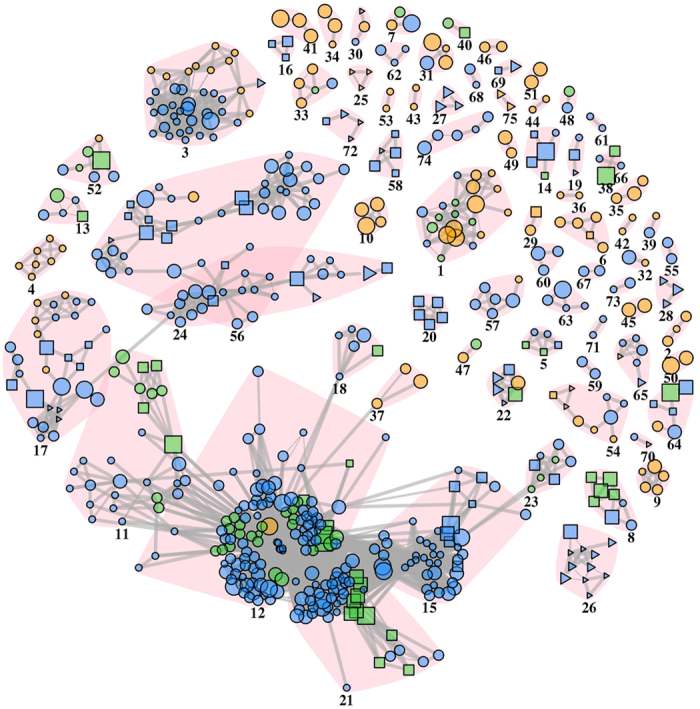
Network of pathways enriched in genes with different somatic mutations and gene expression levels between two PRAD prognostic groups. Vertices are pathways with *p* < 0.003 (GSEA, estimated with permutations) in at least one interactome and links indicate the similarity between pathways (*o* ≥ 0.95); communities of similar pathways are underlined by pink background and identified by numbers (Supplementary Data S3); pathways that are not similar to any other pathway are not shown; green = pathway found in SM and GE data; yellow = SM only; blue = GE only; circle = pathway found only when using network-based quantities (Δ*S* or *Sp*); triangle = pathway found only when using network-free quantities (Δ*f* or lfcp); square = pathway found by network-based quantities and network-free statistics (Supplementary Data S3).

We carried out gene set enrichment analysis (GSEA)^26^ to identify the molecular pathways regulated by genes with high Δ*S* and high *Sp*. We have found a total of 737 pathways with *p* < 0.005 (estimated with 1000 permutations) in at least one interactome, of which 270 in SM, 556 in GE and 89 in common (Table 3 and Supplementary Data S3). Comprehensively, the significant pathways cover the 8 capabilities (also known as hallmarks) acquired during the pathogenesis of cancer^27^ (Supplementary Data S3). The number of pathways found by GSEA with *p* < 0.005 (estimated with 1000 permutations) on gene lists generated using networks are more than those found by GSEA on gene lists ordered by network-free statistics (Table 3). Therefore, the GSEA on network-based statistics determined a more comprehensive enrichment map^28^, the network of pathways clustered in communities on the basis of common genes (Fig. 6). In addition, the enrichment map shows that, apart few exceptions, the majority of pathways found only with network-free statistics are similar to pathways found by the network-based analysis (Fig. 6).

### Comparison with other diffusion-based methods

We used a non parametric method (SAM^29^) to compare quantile normalised (QN), network-smoothed (NP) SM profiles of G5 and G2 (STRING PPIs), analogously to what was done in a recent work^7^. However, due to the sparsity of PRAD SM data, such approach (NP + QN + SAM) produced a gene ranking characterised by a small overlap with Δ*S*. In fact, many genes with a marginal difference of mutations between G5 and G2 were highly ranked by NP + QN + SAM, because these genes had very conserved differences of their quantile normalised, network-smoothed values between G5 and G2 (Supplementary Fig. S8).

We applied the stSVM method^21^ on PRAD GE data (G5 and G2) and STRING PPIs. We calculated *Sp* using the inferential statistics of stSVM (Student’s *t*) and obtained a strong overlap between the top ranking genes ordered by *Sp* and the 97 genes found by stSVM (Supplementary Fig. S8). Interestingly, we observed the highest overlap for *ε* = 1, the value that we had chosen for the analysis of PRAD GE data (see Fig. 4b). The network resampling procedure suggested the presence of 50 significantly connected genes, 38 of which are in common with the 97 genes found by stSVM (Supplementary Fig. S8).

## Discussion

We introduced the network smoothing index (*S*), a network diffusion-based way of interpreting the molecular profiles in the context of an interaction network. The network smoothing index summarises the amount of omics information of an entity jointly with the amount of information of its network neighbourhood, defined considering the whole network topology via network diffusion. The comparison of *S* between two groups of samples (Δ*S*) is a network-based measure that indicates the differential amount of molecular variation and intrinsically mitigates the influence of topology on network smoothed values of the two groups. Alternatively, when the initial statistics is inferential, *S* can be adjusted by means of *p* values estimated with permutations, obtaining *Sp*.

In general, *S*, Δ*S* and *Sp* determine a network-based prioritization of molecular entities that highlights network regions enriched in molecular alterations. For example, such quantities allow: to find altered genes that are also involved in similar biological processes; to discriminate genes with similar molecular profiles, which is especially useful in case of ties; to highlight possible co-players of a pathological process, which have marginal molecular variations but are in network proximity to genes with relevant variations.

The complexity of biological networks makes the precise definition of a network region involved in a biological process or pathology a challenge, and several approximated or heuristics approaches exist to deal with this issue^13^. We showed that the application of network resampling to gene lists sorted by Δ*S* (or *Sp*) suggests possible significantly connected components on the basis of Δ*S* (or *Sp*) values distribution over the network. This procedure gives the opportunity to focus further analyses on any of such components in relation to specific objectives or *a priori* knowledge.

Molecular entities sorted by Δ*S* or *Sp* values can be used as input for further analyses, such as pathway analysis. When used in combination with a method of pathway analysis, like GSEA^26^, Δ*S* or *Sp* allow the quantification of molecular variations occurring in functional modules (pathway-topology based analysis^30^).

We showed that network propagation, after proper rescaling, is equivalent to a physical model that describes the diffusion of a virtual quantity throughout a network^8^,^31^. The connection of the two models allows a better understanding of the meaning of the used parameters and allows a better comparison with similar approaches.

As a proof of principle, we have calculated Δ*S* on somatic mutations and *Sp* on gene expression data from PRAD samples of different prognostic groups (G5 and G2). We showed that Δ*S* and *Sp* highlight, respectively, network regions enriched in a higher content of SM and GE variations of G5 in comparison to G2. We have focused on Δ*S* > 0, but also the opposite or its absolute value can be meaningful, depending on the objective of the analysis. A deeper investigation of PRAD biology is beyond the scope of our work, nevertheless, we provide several genes which are very likely to have a role in the prognostic outcome. In fact, these genes lie in network proximity to genes already associated with PRAD and in PPI network regions enriched in mutated and/or differentially expressed genes. In line with the local hypothesis our analysis revealed the existence of a large connected component of genes that are associated with molecular variations (genetic mutations and/or differential expression) between subjects of different prognostic groups.

The repetition of a network-based analysis using different interactomes is important, because the current reconstructions are still incomplete. We obtained qualitatively similar results and good overlaps using different interactomes, despite intrinsic differences in terms of number of proteins, interactions and topological density. On the other hand, each interactome has its own characteristics, which lead to specific results in terms of genes and biological processes.

## Methods

### Network Diffusion

The adjacency matrix **A** was normalised by dividing each element *a*_*ij*_ by the square root of the product of the degrees (*k*_*i*_, *k*_*j*_) of the corresponding vertices:


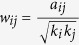


Network propagation (Equation 1) was run iteratively for *t* = [0, 1, 2, …] until convergence: |*X*_*t*+1_ − *X*_*t*_| < 10^−6^. The choice of parameter *α* influences the behaviour of the diffusion algorithm, since *α* controls how much information is kept in vertices versus how much tends to be spread through the network. From a physical point of view it is reasonable to assume that *α* > 0.5, which corresponds to an increase in the importance of network topology. Therefore, *α* was set to 0.7, a value that determined consistent results in previous studies^7^,^20^ and is a good trade off between diffusion rate and computational cost (which increases as *α* → 1).

### Molecular interaction data

Five sources of PPIs data were considered, abbreviated as STRING, NCBI, HI, FP60 and GHIASSIAN. Native identifiers were mapped to Entrez Gene^32^ identifiers using NCBI data released June 26th 2015. STRING interactions were downloaded from STRING (version 10) web site, a database of direct and indirect PPIs^24^; in case multiple proteins mapped to the same gene identifier, only the pair of gene identifiers with the highest STRING confidence score was considered; a total of 11,535 genes and 207,157 links with confidence score ≥700 were retained. NCBI interactions were downloaded from NCBI ftp service, for a total of 15,098 genes and 159,092 links. HI protein links were collected from Rolland *et al.*^33^ and a total of 7,760 genes and 25,040 links were obtained. FP60 interactions were collected from Kotlyar *et al.*^14^ and a total of 10,363 genes and 258,923 links were retained. GHIASSIAN protein interactions were collected from Ghiassian *et al.*^6^, for a total of 13,253 genes and 138,126 links.

### Prostate adenocarcinoma data

PRAD clinical data were downloaded from the TCGA portal^25^. Prognostic grade groups based on the Gleason grading system were calculated as proposed in Pierorazio *et al.*^34^: Gleason score ≤6 (prognostic grade group 1, G1); Gleason score 3 + 4 = 7 (G2); Gleason score 4 + 3 = 7 (G3); Gleason score 4 + 4 = 8 (G4); and Gleason score 9–10 (G5). Groups G2 and G5 contained respectively 110 and 41 subjects (Supplementary Data S4).

Prostate adenocarcinoma (PRAD) curated somatic mutation (SM) data (collected with the Illumina Genome Analyzer platform) and PRAD RNA sequencing data (GE) (collected with the Illumina HiSeq 2000 RNA Sequencing (Version 2) platform) were downloaded from the TCGA portal. Only primary solid tumours (TCGA short letter code “TP”) were considered. Both datasets were updated to Entrez Gene^32^ identifiers released June 26th 2015.

SM dataset was composed of a total of 151 subjects (G2 and G5) with mutations in 6,898 genes (subjects with <10 mutations or with >200 were not considered). This dataset was encoded as a binary genes-by-samples matrix where the generic element *a*_*ij*_ was set to 1 if the patient *j* had at least one mutation in gene *i*, analogously to Hofree *et al.*^7^. Then gene mutation relative frequencies were calculated for each prognostic group.

Multiple gene expression profiles mapped to the same gene were collapsed considering the “MaxMean” criterion (implemented in the WGCNA^35^ R package). Only genes with more than 5 counts in at least 25% of subjects were considered. The dataset was normalized using the TMM method (trimmed mean of M values^36^) available in edgeR^37^ R package, and log-cpm (count-per-milion) values were obtained using the “voom” function available in limma^38^ R package. Only genes with cpm >1 in at least 25% of subjects were considered. A total of 14,676 genes and 151 subjects (G2 and G5) were obtained. A vector of differential expression statistics was calculated from fold changes (*FC*) between G5 and G2, and the corresponding *p* values adjusted for false discovery rate (from limma^38^), as described in Xiao *et al.*^39^: *lfcp *= −log_10_ (*p*)|log_2_ (*FC*)|.

### Simulated datasets

Simulated modules were defined as random subnetworks of the STRING^24^ PPI network, as previously described in Mosca *et al.*^20^. Briefly, a “seed” gene is randomly selected and, then, up to 5 direct interactors are added to the current module. This procedure is repeated randomly selecting a new seed among the current module genes until the desired module size is reached. Note that this procedure defines connected subnetworks with different topological features (modularity, clustering coefficient, etc.).

The vector of gene SM relative frequency (*f*) was permuted, such that the initial sums of SM per patient and per gene across all subjects were not modified. At this point, gene labels were re-assigned in order to obtain the desired frequencies on the module. The re-assignment is controlled by the two parameters *m*_%_, the percentage of mountains (genes with the highest frequencies) within the module, and *h*, the average frequency of hills (genes with lower frequencies) (Fig. 2a). We define the fraction of “signal” (*ω*) associated with a module as:


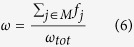


where 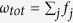. Therefore for any fixed value of *m*_%_, *ω* increases with *h* and *M* (Supplementary Fig. S9).

The recall was defined as the fraction of the top ranking genes, sorted by decreasing order of *S*, which belong to the module of size *M*: 
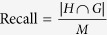
, where *H* is the set of the first *M* genes ranked by decreasing order of *S* and *G* are module genes.

### Pathway analysis

Pathway analysis was carried out using the gene set enrichment analysis approach^26^. Genes were ranked in decreasing order of Δ*S* or *Sp*. NCBI Biosystems^40^ was used as source of gene-pathway associations; only pathways with a number (*n*) of genes 10 ≤ *n* ≤ 300 were considered. Enrichment scores and associated *p* values were calculated by means of the HTSAnalyzeR^41^ R package using 999 permutations. The *p* values calculated by HTSAnalyzeR were updated according to the equation *p*′ = (*p* ⋅ 999 + 1)/1,000, in order to count the real gene ranking as one among the 1,000 permutations. The similarity between two gene sets (*A*, *B*) was calculated using the overlap coefficient: 
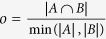
.

### Data mining of PRAD literature

Literature-based text mining was performed using ProteinQuest (PQ) (http://www.proteinquest.com). PQ is a web based platform for biomedical literature retrieval and analysis. PQ searches within PubMed abstracts and image captions from free full text articles. PQ text-mining tool parses target documents searching for terms related to curated ontologies (e.g. diseases, bioprocesses, pathways, body parts). Multiple searches for more than one alias were used to resolve ambiguities in the terminology. PQ was queried in order to retrieve the co-occurrence of genes and PRAD in the scientific literature (Supplementary Data S5).

### Other diffusion-based methods

Network smoothed somatic mutation profiles were quantile normalised with the normalizeQuantiles function of limma^38^ R package. SAM statistics were computed with the samr (http://CRAN.R-project.org/package=samr) R package, using parameters “Two class unpaired” and “wilcoxon”. The netClass^42^ R package was used as implementaton of stSVM^21^.

## Additional Information

**How to cite this article**: Bersanelli, M. *et al.* Network diffusion-based analysis of high-throughput data for the detection of differentially enriched modules. *Sci. Rep.* **6**, 34841; doi: 10.1038/srep34841 (2016).

## Supplementary Material

Supplementary Information

Supplementary Data S1

Supplementary Data S2

Supplementary Data S3

Supplementary Data S4

Supplementary Data S5

## Figures and Tables

**Table 1:** Module size and common genes across interactomes.

Interactome (genes)	SM module	GE module
FP60 (10,363)	109	100
GHIASSIAN (13,253)	117	351
HI (7,760)	231	308
NCBI (15,098)	117	100
STRING (11,535)	126	177
*n* ≥ 1	342	518
*n* ≥ 2	132	257
*n* ≥ 3	104	144
*n* ≥ 4	77	84
*n* = 5	45	33

*n* = number of interactomes.

**Table 2:** Ranking of module genes with the highest occurrence in the literature of PRAD.

Symbol	SM		GE		Occurrence
	Δ*S*	Δ*f*	*Sp*	lfcp	
TP53	1	1	155	2401	2075
PIK3CA	85	104.5	—	—	935
BIRC5	—	—	24	361	589
PTGS2	30	64	—	—	465
EZH2	—	—	57	412	395
CDK2	—	—	172	3324	391
CDK1	—	—	1	83	380
BRCA2	82	125.5	—	—	376
E2F1	—	—	44	235	305
CCNB1	—	—	174	639	284
CC-2	—	—	21	222	239
SERPINB5	—	—	67	151	239
CBX2	—	—	45	154	201
SMAD4	20	64	—	—	139
MEFV	73	707.5	—	—	81
HDAC6	42	64	—	—	48
CHD1	55	64	—	—	29
TRPS1	119	707.5	—	—	26
IDH1	14	16.5	—	—	14
MST1R	39	64	—	—	13

The occurrence is reported as number of papers; the symbol “—” indicates genes not included in gene modules; these results are relative to STRING PPIs (Supplementary Data S1–2).

**Table 3:** Number of significant pathways.

	Network-free	Network-based	∩	∪
SM	27	270	9	288
GE	151	556	95	612

∩ = interesection; ∪ = union.
