# Supplementary material for: Network diffusion-based analysis of high-throughput data for the detection of differentially enriched modules
Source: Sci Rep. 2016 Oct 12;6:34841. doi: 10.1038/srep34841 (PMC5059623; doi:10.1038/srep34841)
Supplement: Supplementary Information [file srep34841-s1.pdf]

Network diffusion-based analysis of high-throughput data for the detection of differentially enriched modules

Matteo Bersanelli[1+], Ettore Mosca[2+*], Daniel Remondini[1], Gastone Castellani[1] and Luciano Milanesi[2]

[1] Department of Physics and Astronomy, Universita' di Bologna, Bologna, via B. Pichat 6/2, 40127, Italy
[2] Institute of Biomedical Technologies-CNR, Segrate(MI), via Fratelli Cervi 93, 20090, Italy
[*]ettore.mosca@itb.cnr.it
[+]these authors contributed equally to this work

Supplementary Notes and Figures

Supplementary Note 2

Supplementary Figure S1: Network resampling p value 5

Supplementary Figure S2: The choice of ϵ in PRAD SM data 6

Supplementary Figure S3: The choice of ϵ in PRAD GE data 7

Supplementary Figure S4: Comparison of network-based and network-free quantities on FP60 PPIs. 8

Supplementary Figure S5: Comparison of network-based and network-free quantities on GHIASSIAN PPIs. 9

Supplementary Figure S6: Comparison of network-based and network-free quantities on NCBI PPIs. 10

Supplementary Figure S7: Comparison of network-based and network-free quantities on HI PPIs. 11

Supplementary Figure S8: Comparison with other diffusion-based methods 12

Supplementary Figure S9: Relationship between ω and h 13

Supplementary Note

Network diffusion

We are interested in studying the the stationary distributions X_* dependence on the altered nodes that for each sample are summarized by the vector X_0 where the i-th component is 1 if the i-th molecular entity is altered and 0 otherwise. We consider the network propagation equation

$$X_{t+1} = \alpha W \cdot X_t + (1-\alpha)X_0 \tag{1}$$

In equation (1) during each iteration each node receives the information from its neighbors, and also retains its initial information and self-reinforcement is avoided. Moreover the information is spread symmetrically since W is a symmetric matrix. The algorithm convergence is demonstrated using the power extension method; we set $X_0 = X(0)$ and we apply some iterations:

$$\begin{aligned} X_1 &= \alpha W X_0 + (1-\alpha)X_0, \\ X_2 &= \alpha W X_1 + (1-\alpha)X_0 \\ &= \alpha W(\alpha W X_0 + (1-\alpha)X_0) + (1-\alpha)X_0 \\ &= (\alpha W)^2 X_0 + (1-\alpha)(\alpha W + I)X_0 \\ &= (\alpha W)^2 X_0 + (1-\alpha)((\alpha W) + (\alpha W)^0)X_0 \end{aligned}$$

Iterating this procedure at step t we get:

$$X_t = (\alpha W)^t X_0 + (1-\alpha)\sum_{i=0}^{t-1}(\alpha W)^i X_0,$$

Since $0 < \alpha < 1$ and the eigenvalues of W are in $[-1; 1]$, when we take the limit for $t \to \infty$ we get:

$$X_* = (1-\alpha)(I - \alpha W)^{-1} X_0 \tag{2}$$

This method is successfully exploited by Hofree *et al.* [1] in their network based approach applied to somatic mutation profiles. We now try to give a physical interpretation of such a diffusive algorithm in order to be able to inherit some helpful concepts from an associated physical model.

Physical model

The network propagation algorithm can be interpreted as the discrete implementation of a continuous linear dynamical system; we define $L_\alpha = I - \alpha W$ the symmetrically normalized Laplacian matrix with the perturbation parameter α; and consider the following system of ODEs written in vector form:

$$\dot{X} = -L_\alpha X + (1-\alpha)X_0 \tag{3}$$

We can rewrite equation (3) by defining a new sink parameter $\gamma > 0$ so that $\gamma = \frac{1-\alpha}{\alpha}$ so that, after rescaling time by the parameter α:

$$\dot{X} = -\left(L + \gamma I\right)X + \gamma X_0 \tag{4}$$

where $L = I - W$ is the symmetrically normalized Laplacian matrix. Equation (4) is essentially the physical model cited by Vandin [3] for defining a gene prioritization algorithm on the basis of the work of Qi et al. [2]; Qi defines a continuous-time model for the distribution of a hypothetical fluid in the network. All nodes contain no fluid initially. Query nodes are then selected to serve as sources, where the fluid is pumped in at a constant rate. Fluid diffuses from node to node through the network according to the edge connections. The source imput is balanced by fluid loss out of each node at the constant first order rate γ. The stationary solution of the dynamical system is of course equation (2) and, expressed in terms of the constant sink parameter γ:

$$X_* = \gamma(L + \gamma I)^{-1} X_0 \tag{5}$$

The choice of α and γ

The choice of parameter α (or equivalently γ) influences the behavior of the diffusive algorithm since α controls how much information is retained in the nodes versus how much tends to be spread in the network. From a physical point of view it is reasonable to assume that $\alpha > 0.5$, in order to make network topology relevant. Throughout the analysis of several real or toy datasets we find that the importance of the choice of a specific value of $0.5 \leq \alpha < 1$ is somehow negligible, in the sense that the results are very similar for different choices of α. However qualitatively it is a good trade off between diffusion rate and computational cost (which increases as $\alpha \to 1$) to take the parameter $\alpha = 0.7$.

Discussion

The connection of the discrete model described by equation (1) to the continuous model allows us to point out that we are dealing with an open system in which the amount of information in each node depends on the constant rate of the sinks γ and on the query nodes distribution. The system is open in the sense that the conservation of fluid amount from a macroscopic point of view and the probability conservation from a microscopic point of view is not guaranteed. The non conservative system shows that X_* represents the quantity of fluid remaining after the flow stabilizes; in each node the fluid pumped by the altered nodes is balanced by the constant sinking of the fluid at the constant rate γ. In principle also the amount of overall fluid on the network could differentiate from cases to controls but we observe that on large networks differences in this sense are not sensible; on the other hand the fact that we are dealing with an open system implies that the final overall amount of fluid depends on both the distribution and the number of initially altered species. Hofree et al [1] normalize each diffused profile so that the sum of the fluid in each sample is constant therefore actually interpreting equation (1) as a random walk with restart. In our work we chose not to normalize each profile in this fashion, but we use the non-normalized profiles to define the S scores and the ΔS scores where the actual amount of fluid on each node is taken into account. This allows to better highlight the nodes that gain more fluid in one class versus the other even in patients in the same class that have a different number of altered entities.

Network resampling procedure

The definition of network resampling p values (p_{nr}) is performed by comparing the objective function Ω with a sample of its local perturbations Ω_σ. We first define, as in the main text, the objective function Ω:

$$\Omega(n) = \Delta S^t(n) \cdot \mathbf{A}_n \cdot \Delta S(n) \tag{6}$$

where $\mathbf{A}_n$ is the adjacency matrix between the first n top scoring entities and $\Delta S(n)$ are the first n scores decreasingly ordered. Equation (6) can be seen as a scalar product between the top highest ΔS , where the matrix A_n makes sure that only the scores associated with entities that have at least a connection to the remaining $n-1$ ones positively contribute to the calculation. The Ω function is non-decreasing since only positive scores are considered. A single perturbation of function (6) is defined as follows:

$$\Omega_{\sigma(n)} = \Delta S^t(n) \cdot \mathbf{A}_{\sigma(n)} \cdot \Delta S(n) \tag{7}$$

where $\sigma(n)$ is a random permutation between the first n molecular entities labels, so that $\mathbf{A}_{\sigma(n)}$ is simply a random resampling of the existing connections between the top scoring genes. We specify that the permutations are constructed as follows: we first randomly permute the indexes of all the genes and then we use that random reassignment to define a single $\Omega_{\sigma(n)}$ perturbation; in this fashion we construct perturbations that behave similarly to equation (6), with the advantage of a graphical feedback (for example, see Supplementary Fig. S3).

In order to define network resampling p-values we produce a set of k perturbations and for each value n we compute the fraction of times in which the perturbations are above or equal to the objective function Ω. As log as for some of the k permutations it holds that $\Omega_\sigma(n) \geq \Omega(n)$ it means that the first n top scoring genes are connected enough that a resampling of the connections among them do not alter too much the strength of the subnetwork, while it's

reasonable to expect a sensible deviation of the permutations from the objective function when top-scoring genes that are not connected to the previous $n-1$ ones enter the top of the list. Therefore for each value n we take k different permutations of the indexes $\{\sigma_1, \sigma_2, \cdots, \sigma_k\}$ compute:

$$p_{nr}(n) = \frac{|\{j \in (1, \cdots, k) \ \mid \ \Omega_{\sigma_j}(n) \geq \Omega(n)\}| + 1}{k+1} \qquad (8)$$

where we add 1 both at the numerator and at the denominator so that the smallest p value is never null. At this point, according to the model's assumptions, in principle any local minimum of equation (8) could be an interesting choice for cutting the top of the ΔS list; however in our applications we find reasonable to cut at the first local minimum $\hat{p}_{nr}$ since it often represents the value corresponding to the value $\bar{n}$ where the perturbations (equation (7)) start to sensibly deviate from the objective function (equation (6)). See supplementary Fig. S3.

Supplementary Figure S1: Network resampling p value

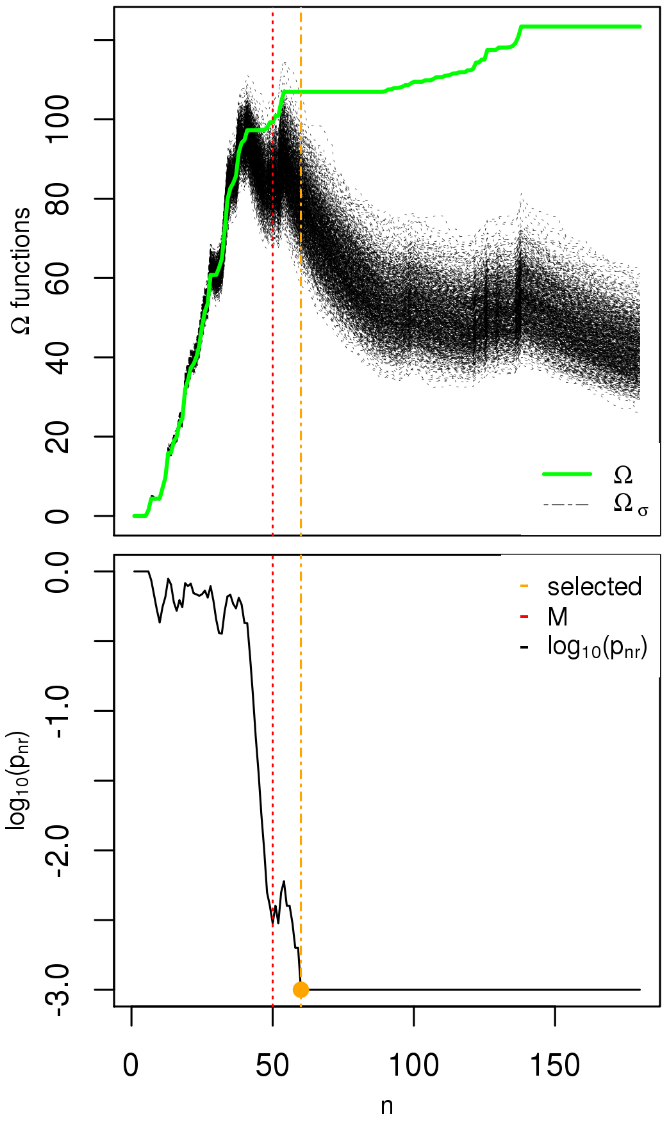

The deviations of 999 permutations Ω_σ from the objective function Ω (top) captures the biological signal within the synthetic module of size $M = 50$. Such deviation is captured by the first minimum p_{nr}-value (bottom).

Supplementary Figure S2: The choice of ϵ in PRAD SM data

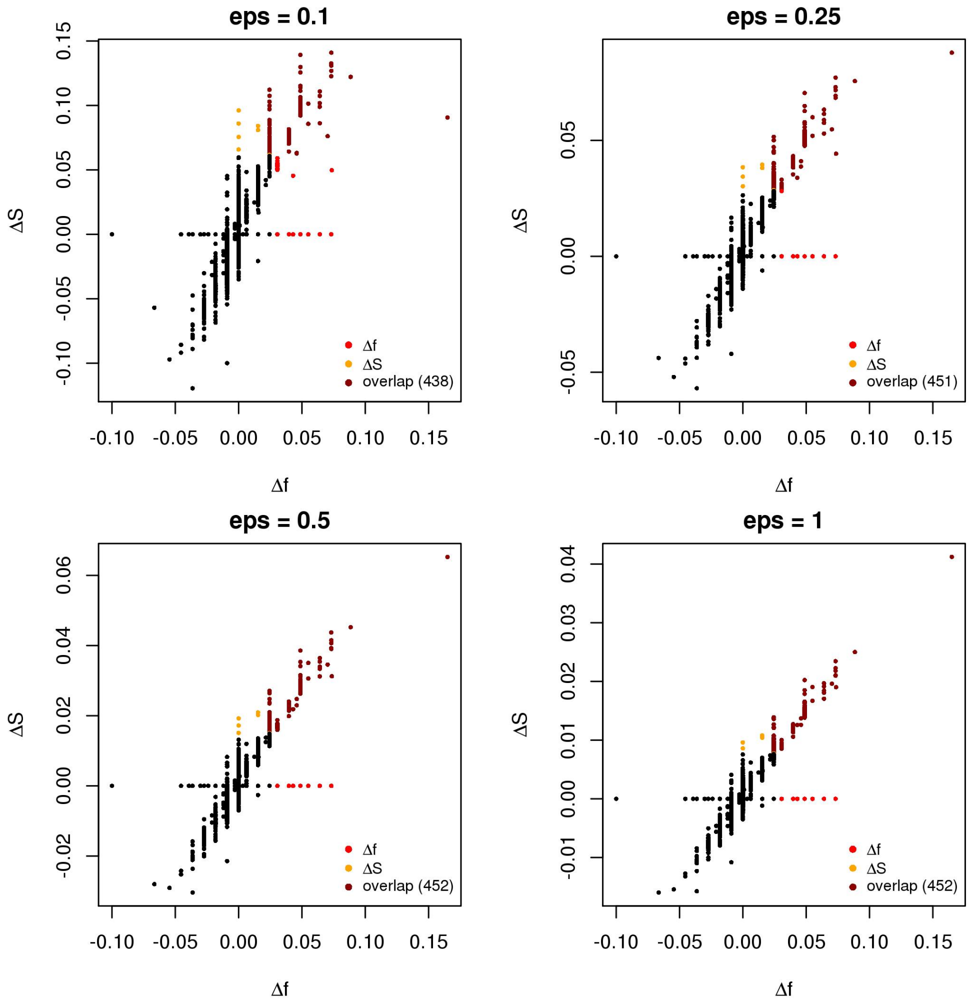

Scatter plot with Δf values and ΔS calculated on PRAD SM data for different values of the parameter ϵ. The overlap is calculated over the top 500 genes of the two quantities on STRING PPIs.

Supplementary Figure S3: The choice of ϵ in PRAD GE data

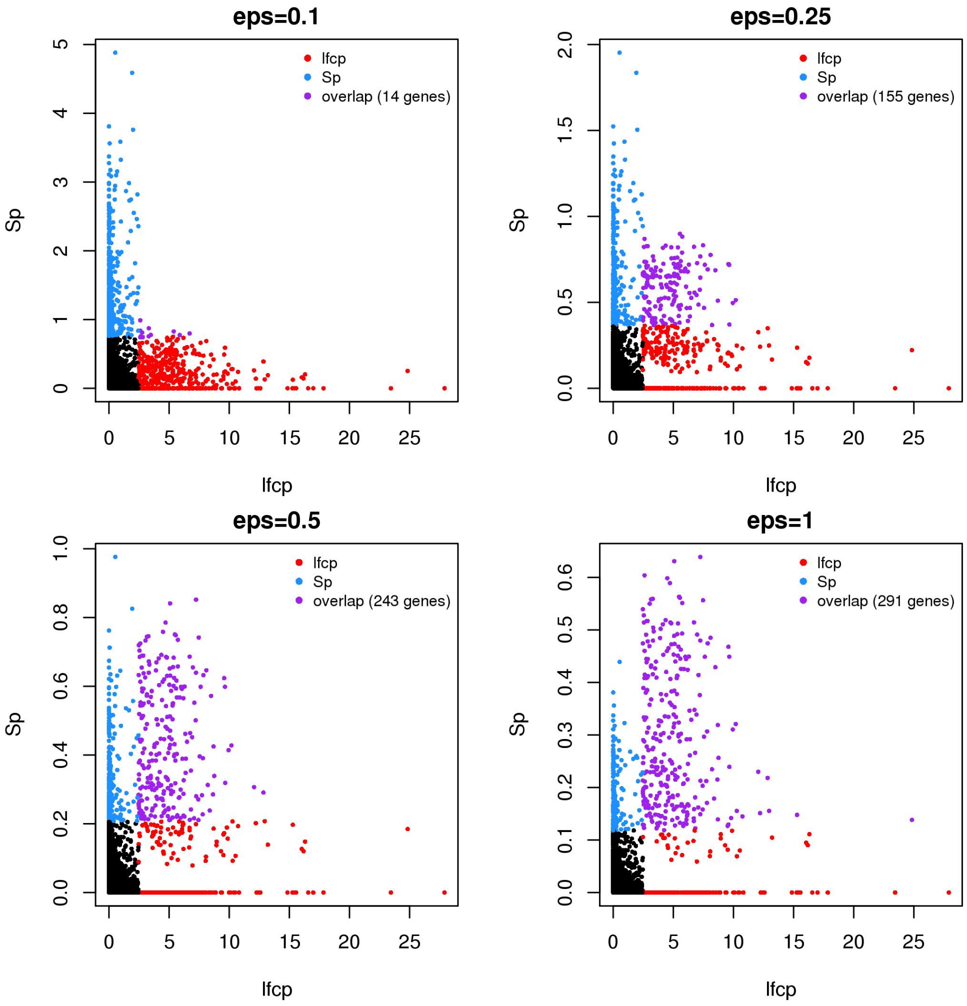

Scatter plot with $lfcp$ values and Sp calculated on PRAD GE data for different values of the parameter ϵ. The overlap is calculated over the top 500 genes of the two quantities on STRING PPIs.

Supplementary Figure S4: Comparison of network-based and network-free quantities on FP60 PPIs.

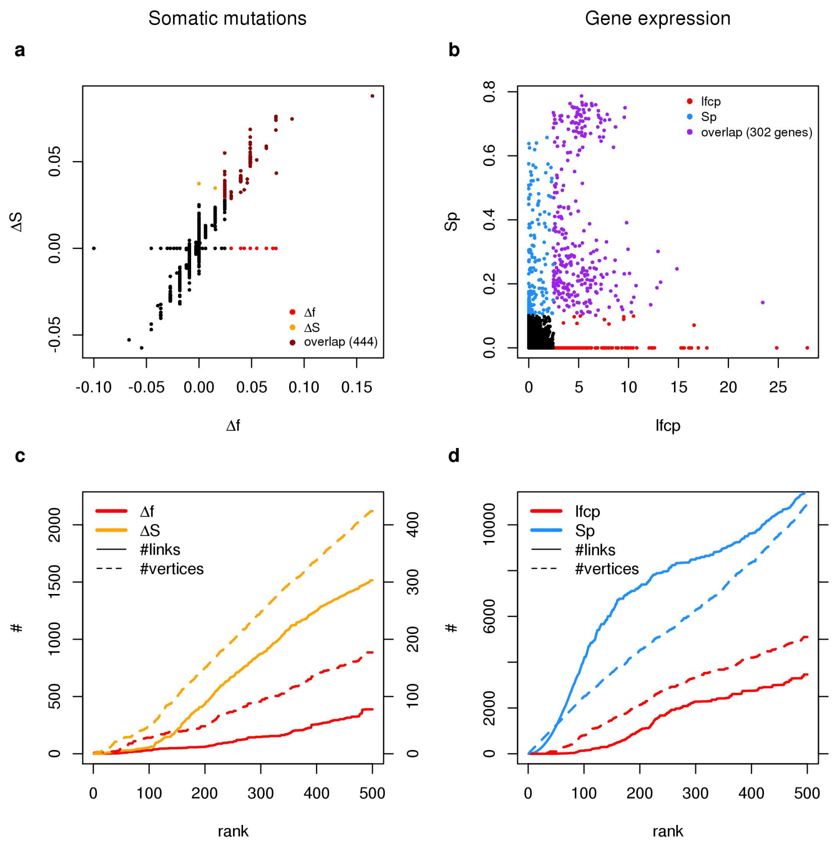

Comparison of network-based and network-free quantities calculated on somatic mutation and gene expression data from PRAD samples associated with two different prognostic groups. (a-b) Scatter plot with network-based ($y-$axis) *vs* network-free ($x-$axis) gene scores calculated on PRAD SM (a) and GE (b) data; colours indicate the top 500 genes ranked by network-free (red) or network-based (yellow, blue) scores and the overlaps (brown, purple). **(c-d)** Number of links ($y-$axis, left) and number of connected genes ($y-$axis, right) within the first 500 genes ordered by network (ΔS, Sp) and network-free (Δf, $lfcp$) gene scores, calculated on PRAD SM (c) and PRAD GE (d) data. **(a-d)** ΔS and Sp were calculated using FP60 PPIs and, respectively, $\epsilon = 0.25$ and $\epsilon = 1$. **(c-d)** #: number of links (vertical axis, left) or number of veritces (vertical axis, right).

Supplementary Figure S5: Comparison of network-based and network-free quantities on GHIASSIAN PPIs.

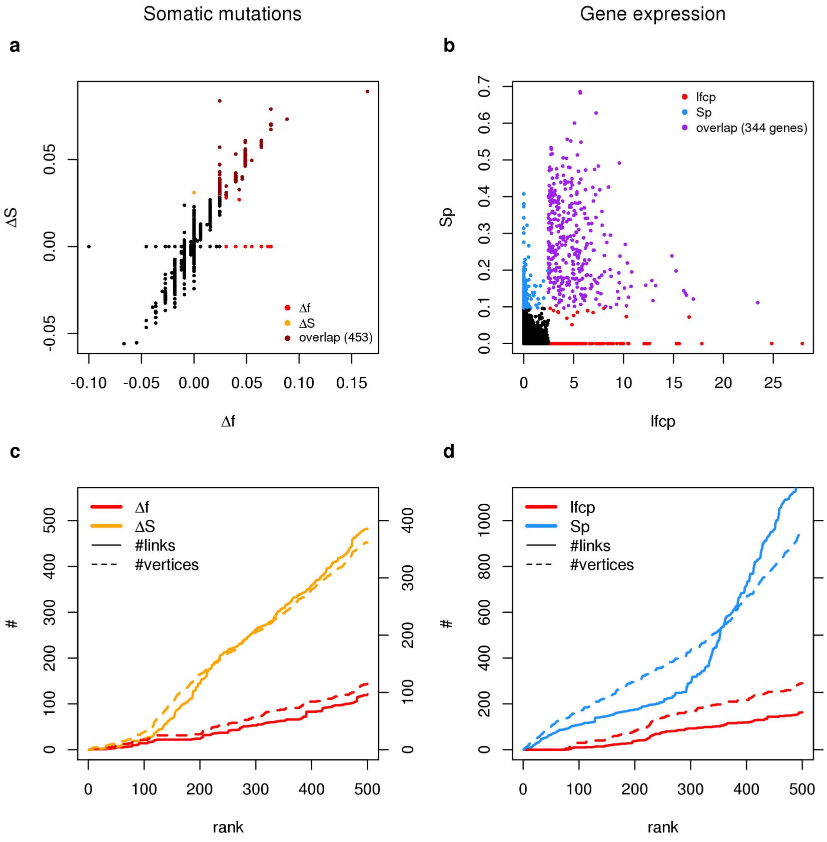

Comparison of network-based and network-free quantities calculated on somatic mutation and gene expression data from PRAD samples associated with two different prognostic groups. (a-b) Scatter plot with network-based ($y-$axis) *vs* network-free ($x-$axis) gene scores calculated on PRAD SM (a) and GE (b) data; colours indicate the top 500 genes ranked by network-free (red) or network-based (yellow, blue) scores and the overlaps (brown, purple). **(c-d)** Number of links ($y-$axis, left) and number of connected genes ($y-$axis, right) within the first 500 genes ordered by network (ΔS, Sp) and network-free (Δf, $lfcp$) gene scores, calculated on PRAD SM (c) and PRAD GE (d) data. **(a-d)** ΔS and Sp were calculated using GHIASSIAN PPIs and, respectively, $\epsilon = 0.25$ and $\epsilon = 1$. **(c-d)** #: number of links (vertical axis, left) or number of veritces (vertical axis, right).

Supplementary Figure S6: Comparison of network-based and network-free quantities on NCBI PPIs.

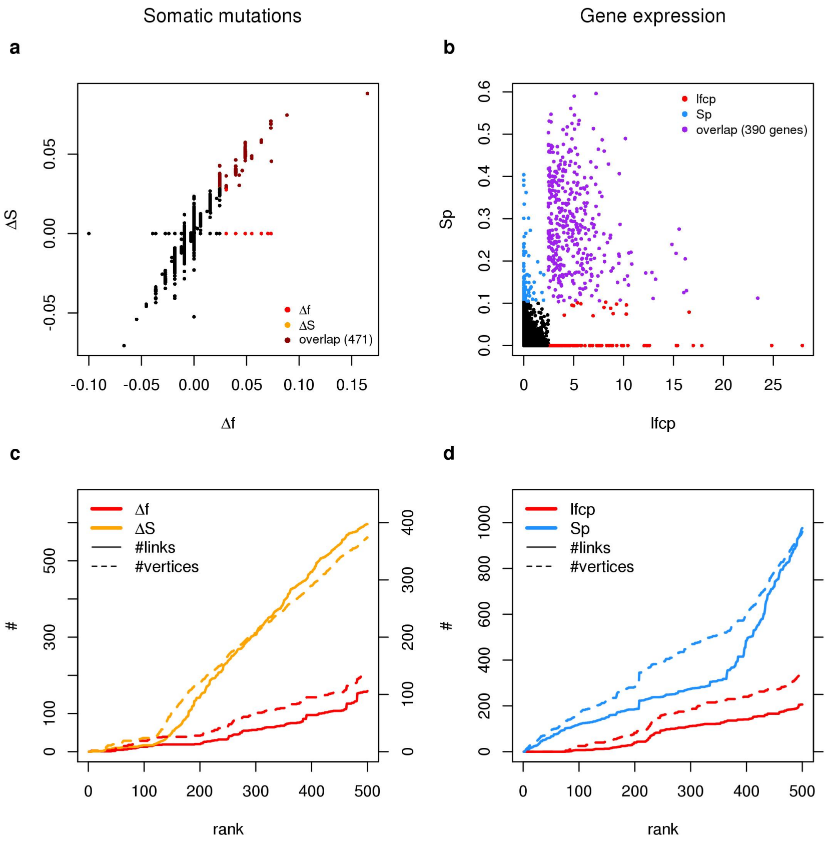

Comparison of network-based and network-free quantities calculated on somatic mutation and gene expression data from PRAD samples associated with two different prognostic groups. **(a-b)** Scatter plot with network-based ($y-$axis) vs network-free ($x-$axis) gene scores calculated on PRAD SM (a) and GE (b) data; colours indicate the top 500 genes ranked by network-free (red) or network-based (yellow, blue) scores and the overlaps (brown, purple). **(c-d)** Number of links ($y-$axis, left) and number of connected genes ($y-$axis, right) within the first 500 genes ordered by network (ΔS, Sp) and network-free (Δf, $lfcp$) gene scores, calculated on PRAD SM (c) and PRAD GE (d) data. **(a-d)** ΔS and Sp were calculated using NCBI PPIs and, respectively, $\epsilon = 0.25$ and $\epsilon = 1$. **(c-d)** #: number of links (vertical axis, left) or number of veritces (vertical axis, right).

Supplementary Figure S7: Comparison of network-based and network-free quantities on HI PPIs.

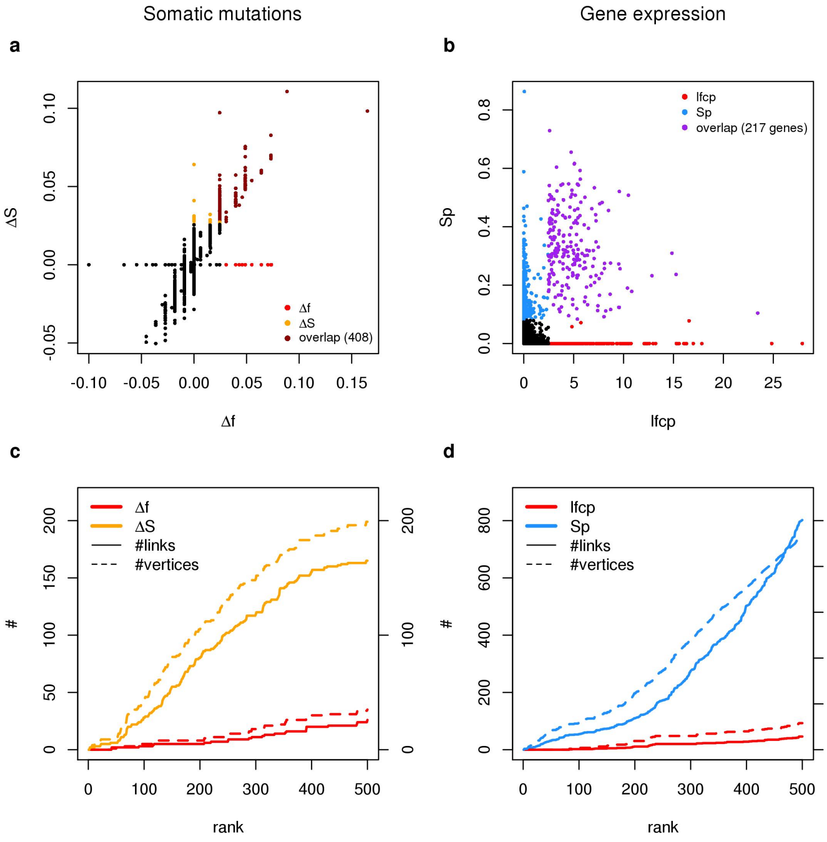

Comparison of network-based and network-free quantities calculated on somatic mutation and gene expression data from PRAD samples associated with two different prognostic groups. (a-b) Scatter plot with network-based ($y-$axis) *vs* network-free ($x-$axis) gene scores calculated on PRAD SM (a) and GE (b) data; colours indicate the top 500 genes ranked by network-free (red) or network-based (yellow, blue) scores and the overlaps (brown, purple). **(c-d)** Number of links ($y-$axis, left) and number of connected genes ($y-$axis, right) within the first 500 genes ordered by network (ΔS, Sp) and network-free (Δf, $lfcp$) gene scores, calculated on PRAD SM (c) and PRAD GE (d) data. **(a-d)** ΔS and Sp were calculated using HI PPIs and, respectively, $\epsilon = 0.25$ and $\epsilon = 1$. **(c-d)** $\#$: number of links (vertical axis, left) or number of veritces (vertical axis, right).

Supplementary Figure S8: Comparison with other diffusion-based methods

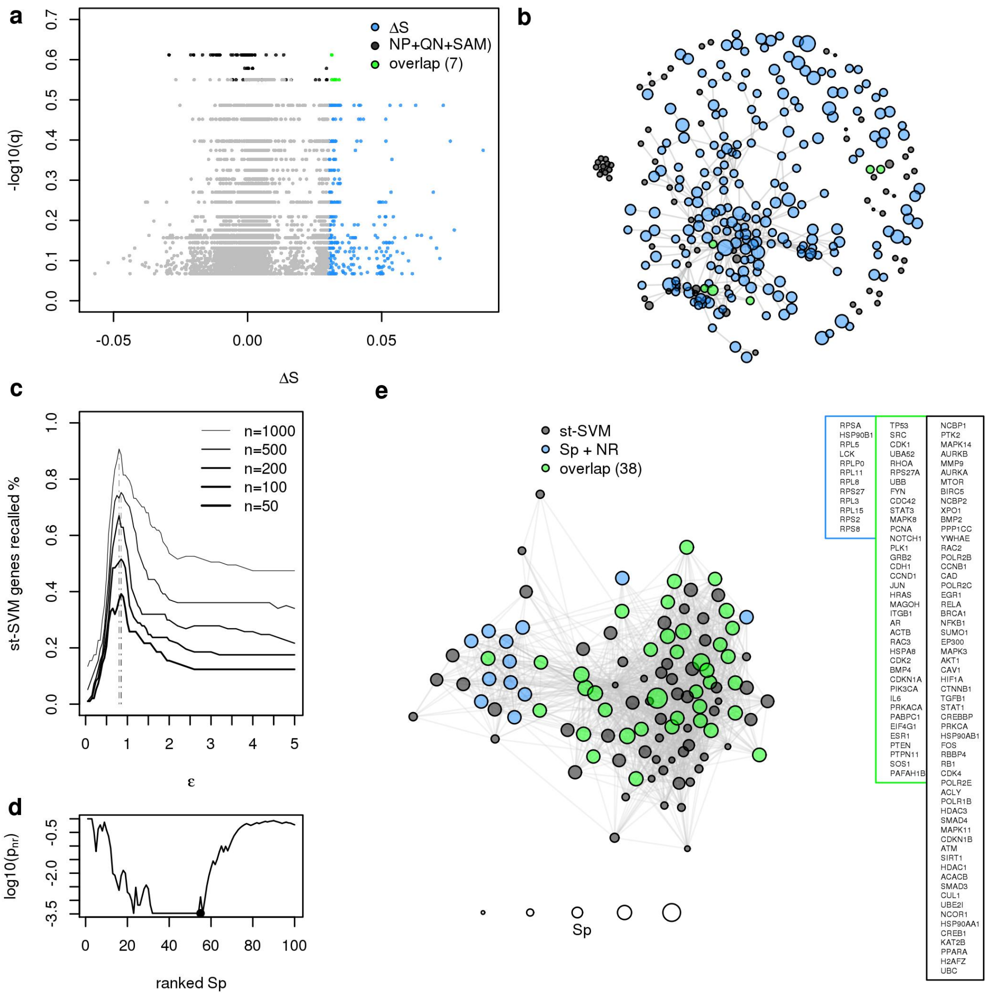

(a) Scatter plot with SAM q values and ΔS calculated on PRAD SM data. **(b)** Network of STRING PPIs formed by the top 300 genes order by SAM q or ΔS; **(c)** Percentage of st-SVM extracted genes recalled on top of the list (from 50 to 1000) of Sp. **(d)** Network resampling applied to the Sp array suggests to cut around 55 top scoring genes. **(e)** Overlap between st-SVM and our method on STRING PPIs.

Supplementary Figure S9: Relationship between ω and h

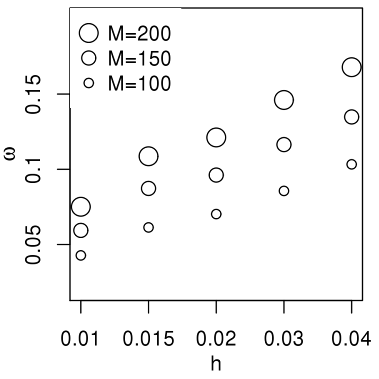

The signal ω as a function of average hills frequency h. The mountains percentage is fixed at $m_{\%} = 0.1$. Values are averaged over ensembles of modules of the same size M.

References

1. Hofree, M., Shen, J. P., Carter, H., Gross, A. & Ideker, T. Network-based stratification of tumor mutations. *Nat. Methods* **10**, 1108–1115 (2013).

2. Qi, Y., Suhail, Y., Lin, Y. Y., Boeke, J. D. & Bader, J. S. Finding friends and enemies in an enemies-only network: a graph diffusion kernel for predicting novel genetic interactions and co-complex membership from yeast genetic interactions. *Genome Res.* **18**, 1991–2004 (2008).

3. Vandin, F., Upfal, E. & Raphael, B. J. Algorithms for detecting significantly mutated pathways in cancer. *J. Comput. Biol.* **18**, 507–522 (2011).
